# A Novel Diagnostic Method to Detect Duck Tembusu Virus: A Colloidal Gold-Based Immunochromatographic Assay

**DOI:** 10.3389/fmicb.2018.01001

**Published:** 2018-05-15

**Authors:** Guanliu Yu, Xianglong Yu, Guoping Yang, Yi Tang, Youxiang Diao

**Affiliations:** ^1^College of Animal Science and Technology, Shandong Agricultural University, Tai’an, China; ^2^Shandong Provincial Key Laboratory of Animal Biotechnology and Disease Control and Prevention, Shandong Agricultural University, Tai’an, China; ^3^Shandong Provincial Engineering Technology Research Center of Animal Disease Control and Prevention, Shandong Agricultural University, Tai’an, China

**Keywords:** DTMUV, monoclonal antibody, virus detection, colloid gold, ICS

## Abstract

Duck Tembusu virus (DTMUV) is an emerging pathogenic flavivirus that has resulted in large economic losses to the duck-rearing industry in China since 2010. Therefore, an effective diagnostic approach to monitor the spread of DTMUV is necessary. Here, a novel diagnostic immunochromatographic strip (ICS) assay was developed to detect DTMUV. The assay was carried out using colloidal gold coated with purified monoclonal antibody A12D3 against envelope E protein. Purified polyclonal C12D1 antibodies from BALB/c mice against the envelope E protein were used as the capture antibody. Goat anti-mouse IgG was used to detect DTMUV, which was also assembled on the ICS. Results showed that the ICS could specifically detect DTMUV within 10 min. It also could be stored 25 and 4°C for 4 and 6 months, respectively. The sensitivity of the ICS indicated that the dilution multiples of positive allantoic fluid of DTMUV (LD50: 10^4.33^/0.2 ml) was up to 200. Its specificity and sensibility showed no significant change under the above storage situations. Fifty clinical samples were simultaneously detected by ICS and reverse-transcription polymerase chain reaction with a 93.9% coincidence rate between them. It proved that the ICS in the present study was highly specific, sensitive, repeatable, and more convenient to rapidly detect DTMUV in clinical samples.

## Introduction

Like other flaviviruses, Duck Tembusu virus (DTMUV) from the *Flaviviridae* family has a 10,990-nt positive-sense single-stranded RNA genome and belongs to the Ntaya antigenic group. The virus was initially isolated from *Culex tritaeniorhynchus* mosquitoes in Malaysia in 1957 and subsequently detected in Thailand and numerous Southeastern Asian areas (Homonnay et al., 2014; Chakritbudsabong et al., 2015; Thontiravong et al., 2015). In China, it was originally detected in Shanghai in April 2010 and then spread rapidly throughout most duck-producing provinces such as Guangdong, Fujian, Jiangsu, Anhui, Henan, and Shandong (Su et al., 2011; Tang et al., 2013). This virus can cause retarded growth, high fever, loss of appetite, severe duck-drop syndrome, and death of the ducks (Yan et al., 2011). Over 10 million ducks have been infected, and more than 1 million ducks died in 2010, which caused massive economic losses in duck-producing industries (Li et al., 2012; He et al., 2017). Moreover, DTMUV poses a potential threat to public health, and it is no longer detectable after the infected fowls recover (Li et al., 2012; Liu et al., 2013; Tang et al., 2013). Therefore, a rapid detection method is necessary to monitor viral occurrence and prevalence.

The colloidal-gold immunochromatography strip (ICS) assay has been widely used for detection of as bacteria (Kong et al., 2017), viruses (Kim et al., 2015; Deng et al., 2017), parasites (Wang et al., 2014), hormones (Nara et al., 2010), and antibodies (Li et al., 2017). ICS uses percolation of the microporous membrane and capillarity, which provides a quick antigen–antibody reaction on the immobilon-P, and then colors for colloidal gold-labeled antibody with a positive reaction appear red. The ICS is suitable for a basic clinical application, which takes a short time and does not require special equipment (Kusano et al., 2014).

Immunochromatographic strip also has been generally applied to detect pathogens in fowls, including the Newcastle disease virus (NDV) (Gao et al., 2004), goose plague virus (Li et al., 2007), avian influenza virus (AIV) (Peng et al., 2008), goose parvovirus (Shaozhou et al., 2015), duck plague virus (DPV) (Zhao et al., 2016), but less applied to DTMUV. Therefore, to satisfy above requirements, an ICS based for E protein was prepared to detect DTMUV from clinical specimens. This study laid the foundation for the further development of a commercial kit.

## Materials and Methods

### Reference Virus Strains and Antibodies

Avian reovirus virus (ARV), H9N2 AIV, DTMUV, DPV, Duck hepatitis A virus type-1 (DHAV-1), egg drop syndrome virus (EDSV), Muscovy duck parovirus (MPV), and NDV reference strains were obtained from our laboratory, the Poultry Disease Lab of Shandong Agricultural University, and used to test the specificity of ICS. The monoclonal antibody, A12D3, against the envelope E protein was produced in our previous study (Ou, 2013), while the polyclonal antibody C12D1 against the envelope E protein was prepared according to the following procedures (**Table 1**). The specificity and sensitivity of above two antibodies were also detected using an indirect ELISA (Supplementary Figures 1, 2; Bai et al., 2015; Lin, 2015). The goat anti-mouse IgG antibody (1 mg/ml) was purchased from Beijing Dingguo Changsheng Biotechnology Co. Ltd., China.

**Table 1:** The immunization procedure for BALB/c mice.

Immunization times	Immunogen	Immunologic adjuvant	Injection route	Dose (μg)
First 7 weeks of age	DTMUV-E protein	Freund’s complete adjuvant	Multiple injection sites in the abdomen hypodermic	100
Second 8 weeks of age	DTMUV-E protein	Freund’s incomplete adjuvant	Multiple injection sites in the abdomen hypodermic	100
Third 9 weeks of age	DTMUV-E protein	Freund’s incomplete adjuvant	Multiple injection sites in the abdomen hypodermic	100
Fourth/strengthen immunity 10 weeks of age	DTMUV-E protein	None	Intraperitoneal injection	80

### Preparation of C12D1

In this study, the polyclonal antibody for C12D1 was prepared by injecting purified DTMUV-E protein into 6 week-BALB/c female mice (purchased from the New Drug Evaluation Center of Shandong University, China) subcutaneously or intraperitoneally following the immunization procedures in **Table 1**. After 1 week, 100 μg of the antigen, E protein, with Freund’s incomplete or complete adjuvant (Becton Dickinson, Franklin Lakes, NJ, United States) was injected intraperitoneally.

After three immunizations per week, the tails were cut on the mice to collect blood samples. E protein (200 ng/well) was used to coat enzyme-labeled plates, and the serum titer was measured as an indirect ELISA (Bai et al., 2015). Mice with a serum titer up to 1:5000 were chosen for the fourth immunization. Blood samples from these mice were collected retro-orbitally in sterilized tubes the third day after the fourth immunization. After leaving the samples undisturbed for 30 min at 37°C, the supernatant was collected and centrifuged at 3,000 rpm for 10 min. The resultant supernatants were used as the crude extracts of C12D1. C12D1 was purified using saturated ammonium sulfate [(NH_4_)_2_SO_4_], and the concentration of C12D1 was determined using an ultraviolet spectrophotometer (Eppendorf BioSpectrometer^®^, Germany) based on the following formula: concentration of protein (mg/ml) = 1.45 × OD_280_ - 0.74 × OD_260_.

### Preparation of the Colloidal Gold Solution

In this study, we prepared colloidal gold solution with an average particle diameter of 20 nm using a tri-sodium citrate reduction method (Liu et al., 2012; Zhao et al., 2016). Before the preparation of the colloidal gold, all glassware used were siliconized with sigmacote, cleaned with aqua regia [HNO_3_/HCL (v/3v)], washed with ultrapure water, and oven-dried (Meng et al., 2014). Subsequently, 1.0 ml of 1% HAuCl_4_ (Aladdin, Shanghai, China) solution was added to 99-ml ultrapure water and boiled thoroughly for 3 min. Then, 2.0 ml of 1% trisodium citrate (Aladdin, Shanghai, China) solution was dropped into the boiled solution, stirred, and heated continuously. When the color of the solution changed from black–blue into red–purple, it was heated for an additional 15 min. Then supplemented ultrapure water was added to the solution until it reached the original volume. Finally, the OD_520_ value of the colloidal gold solution at 500–600 nm was detected by an ultraviolet spectrophotometer (Eppendorf BioSpectrometer^®^, Germany), and the prepared colloidal gold solution was available only when the OD_520_ value was between 0.8 and 1.2 (Wang et al., 2004).

### Optimum pH of the Colloidal Gold Solution

One milliliter of colloidal gold solution was added to eight EP tubes (1.5 ml) and tested with special pH indicator paper, respectively. The pH values were debugged for 6.5, 7.0, 7.5, 8.0, 8.5, 9.0, 9.5, and 10.0 with 0.2 mol/l K_2_CO_3_. After that, 10 μl of A12D3 (1.6 mg/ml) was added into each of the tubes with gentle stirring for 10 min, and then left static for another 10 min at room temperature. 100 μl of NaCl (10%) was added to the above tubes with gentle stirring and left static for 2 h at room temperature. The OD_520_ values were measured using an ultraviolet spectrophotometer at 500–600 nm (Liu et al., 2012; Guo et al., 2017). Finally, the optimum pH of the colloidal gold was analyzed by diagraph.

### Optimum A12D3-Labeled Dose of the Colloidal Gold

One milliliter of optimum pH of colloidal gold solution was added into nine EP tubes, respectively. Then 0–8 μl of A12D3 was added (1.6 mg/ml) into each of the above tubes. After remaining static for 15 min, 100 μl of NaCl (10%) was added to the tubes. Next, after gentle stirring, and then being static for 2 h, the OD_520_ value was measured using an ultraviolet spectrophotometer at 500–600 nm. Finally, diagraph analysis was carried out on the optimum A12D3-labeled dose of the colloidal gold. The stable dose of A12D3 was 1.1–1.2 times the optimum-labeled dose (Hampl et al., 2001).

### Preparation of the Colloidal Gold–A12D3 Conjugate

One milliliter of purified A12D3 (1.6 mg/ml) was added into 50 ml of colloidal gold solution (pH 8.5, slightly more by 0.5 than the optimum pH of colloidal gold solution) with gentle stirring for 1 h. Then, 2.0 ml of 10% of bovine serum albumin (BSA; Beijing Solarbio Science & Technology Co., Ltd., Beijing, China) was added to the solution with gentle stirring for 5 min. Next, 1.0 ml of 10% of PEG2000 (Sigma, United States) was added with gentle stirring for another 5 min. It was centrifuged at 12,000 rpm for 30 min. After centrifugation, the sediment was suspended in 5.0 ml dilution buffer (0.002 mol/l sodium carbonate solution containing 1% BSA and 0.2% sodium azide) and stored at 4°C.

### Preparation of Conjugate Pad

In this study, the colloidal gold–A12D3 conjugate was diluted with 0.02 M PBS containing 1.0% (w/v) BSA and 5.0% (w/v) sucrose at a pH of 8.5. The glass fiber (GL0194; Shanghai JieYi Biotechnology Co. Ltd., China) was incubated with 0.1% Tween-20 for 10 h and dried at 37°C for 2 h. Then, the above colloidal gold–A12D3 conjugate (7.5 μl/cm) was added to the conjugate pad and dried at 37°C for 1 h. It was stored at 4°C for future use.

### Preparation of the Nitrocellulose Membrane

The diluted C12D1 and goat anti-mouse IgG were transferred onto the NC membrane (Millipore, United States) at 1 μl/cm to form the test and the control lines, respectively. The test line was 6 mm apart from the control line. Subsequently, the NC membrane was incubated with blocking buffer containing 1.0% BSA at 37°C for 1 h and then washed two times with wash buffer. The NC membrane was then dried for 2 h at 37°C and stored at 4°C for future use.

### Assembly of the ICS

**FIGURE 1:**
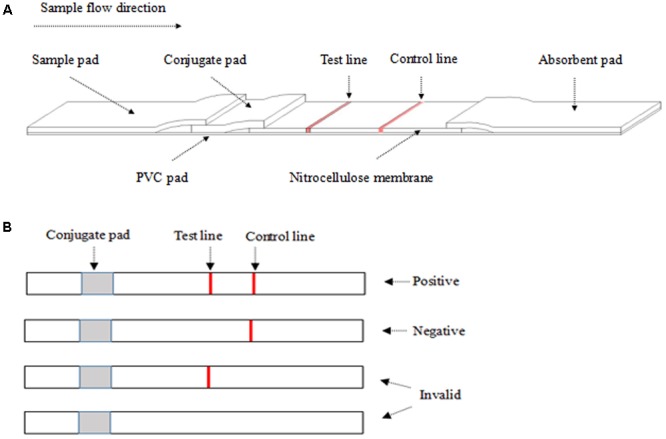
The structure of the colloidal gold test strip **(A)** and the result from ICS **(B)**. **(A)** The liquid sample was added to the sample pad. The test line (polyclonal antibody C12D1) and control line (goat anti-mouse IgG antibody) were examined visually. **(B)** The appearance of two lines indicates a positive result, whereas a valid negative test produces only the control line.

The structure component of ICS is shown in **Figure 1A**. A12D3, C12D1, and goat anti-mouse IgG were coated on the conjugated pad, test line, and control line, respectively. The sample pad, conjugate pad, NC membrane, and absorbent pad were then assembled on the PVC pad (Jiang et al., 2011). Five millimeters of wide strips were cut and then installed in the test card (Liu et al., 2012). Finally, the ICS was stored with desiccant in a sealed bag at 4°C for future use.

### ICS Result

After dropping 100–200 μl of the sample on the sample pad for 10 min at room temperature, a positive result was determined by the appearance of two red lines in the test and the control regions. A negative result was only one red line in the control region. An invalid result was one red line in the test region or no red line on NC membrane (**Figure 1B**).

### Evaluation of the ICS Performance

In this study, to evaluate the performance of ICS objectively, we verified the specificity, sensibility, repeatability, stability, and practicability of prepared ICS, and the details were as follows.

To confirm the specificity of ICS, the positive allantoic fluid samples of DTMUV, NDV, DPV, H9N2 AIV, and ARV were detected at the same time, which were obtained from above virally infected eggs.

For its sensibility, we first diluted the positive allantoic fluid of DTMUV (median lethal dose, LD50 = 10^-4.33^/0.2 ml) in multiples dilution of 10, 50, 100, 200, and 500, and then added the same dose dilution on five ICSs repeated five times.

For the repeatability, we detected the same DTMUV positive allantoic fluid (median tissue culture infective dose, TCID_50_ = 10^1.69^/0.2 ml) with five batches of ICSs (i.e., the ICSs were assembled from the same components at five different stages).

For the stability, we stored the ICS for 10 months at room temperature (25°C) and at 4°C, respectively. During the storage period, we detected the same DTMUV positive allantoic fluid (TCID_50_ = 10^1.69^/0.2 ml) by ICS monthly.

For the coincidence rate of ICS and reverse-transcription polymerase chain reaction (RT-PCR) (Sun et al., 2014; Zhang et al., 2015a; Siu et al., 2016; Zeng et al., 2016), a total of 50 clinical samples (i.e., 35 clinical samples and 15 positive allantoic fluid samples) collected in the field from different duck farms were tested by the ICS. Before detection, the samples were homogenized with an electrical tissue homogenizer (ARTMICCRA D-9, Germany) in PBS containing antibiotics (20% w/v). The sample RNA was extracted with Trizol reagent (TransGen Biotech, Beijing, China) according to the manufacturer’s instruction. The extracted RNA was then used for RT-PCR to evaluate the coincidence rate of the ICS. After the experiment, all of the clinical or viral samples were incinerated.

## Results

### Preparation of C12D1

In this study, the polyclonal antibody for C12D1 was prepared using the immune DTMUV-E protein method. After purification by saturated ammonium sulfate [(NH_4_)_2_SO_4_], the concentration of C12D1 was 1.2 mg/ml.

### Optimization of ICS

**FIGURE 2:**
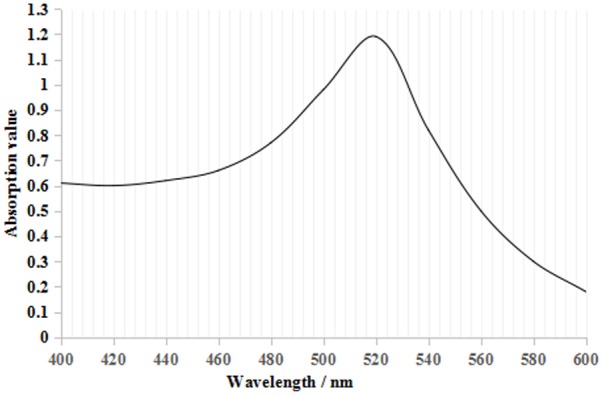
Colloidal gold UV–visible scanning curve. There was only one maximum absorption value at 520 nm, which is the characteristic absorbance peak for colloidal gold.

From **Figure 2**, the maximum absorption wavelength of the colloidal gold solution was 520 nm. According to the following regression equation, *Y* = 0.786*X* + 505.53 (*Y*, maximum absorption wavelength; *X*, particle diameter) (He et al., 2005), the particle diameter of the prepared colloidal gold solution was 18.4 nm. Both parameters satisfied the requirements for colloidal gold preparation (Safenkova et al., 2010; Liu et al., 2012).

**FIGURE 3:**
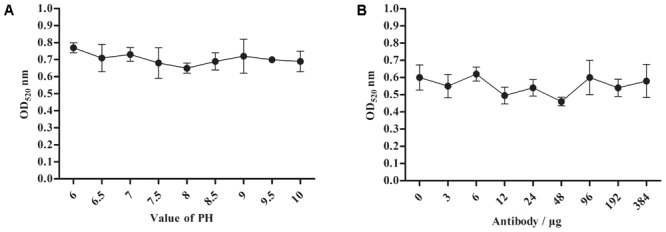
Optimum pH selection for colloidal gold solution **(A)** and a labeled dose selection for A12D3 **(B)**. The optimal pH was 8 and the optimal labeled dose was 48 μg. The error bars correspond to the standard deviations form the data points (*n* = 5).

For the optimum pH and labeled dose of A12D3, we measured the peak values of the solution at the OD_520_ under different pH conditions and labeled doses of A12D3 using an ultraviolet spectrophotometer (Paek et al., 2000; Nara et al., 2010). The lower the OD_520_, the more dispersed the colloidal gold particle was and the more stable the colloidal gold solution was. The optimum pH was 8 (**Figure 3A**), and the optimum of labeled dose of A12D3 was 48 μg (**Figure 3B**).

### Specificity

**FIGURE 4:**
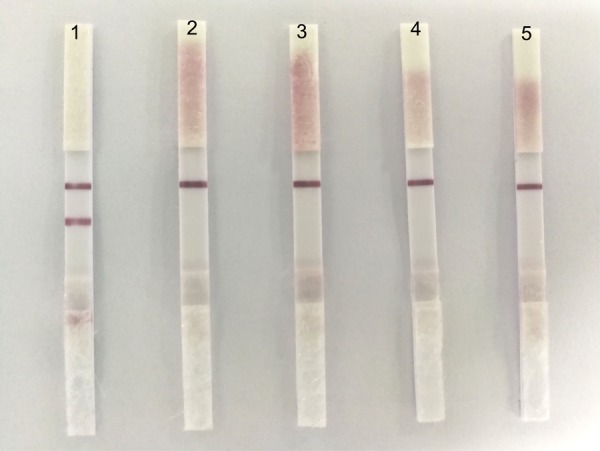
The specificity of the ICS. 1: DTMUV, 2: NDV, 3: DPV, 4: H9N2-AIV, and 5: ARV. ICS only reacted with DTMUV, and there was no cross reaction with NDV, DPV, H9N2-AIV, or ARV.

To determine the specificity of ICS, we tested four usual poultry virus allantoic fluids (i.e., NDV, DPV, H9N2-AIV, and ARV). The results showed that the ICS only reacted to the positive allantoic fluid samples of DTMUV, and had no cross reaction with NDV, DPV, H9N2-AIV, or ARV (**Figure 4**).

### Sensibility

**FIGURE 5:**
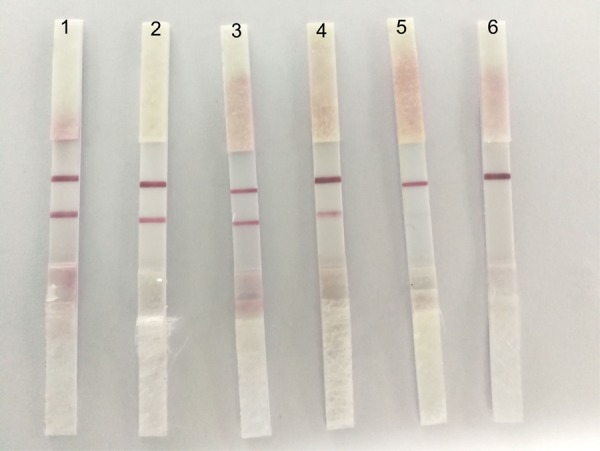
The sensibility of the ICS. The dilution multiples were prepared as follows; 1: 1/10, 2: 1/50, 3: 1/100, 4: 1/200, 5: 1/500, and 6: PBS. A fainter test line appeared at 1:200.

With regard to the sensibility of ICS, the positive allantoic fluid of DTMUV (LD50:10^-4.33^/0.2 ml) was diluted in 10, 50, 100, 200, and 500 dilution multiples, respectively. The results showed that the ICS could detect highly diluted allantoic fluid from DTMUV (LD50: 10^4.33^/0.2 ml) at 1:200 (**Figure 5**).

### Repeatability

**FIGURE 6:**
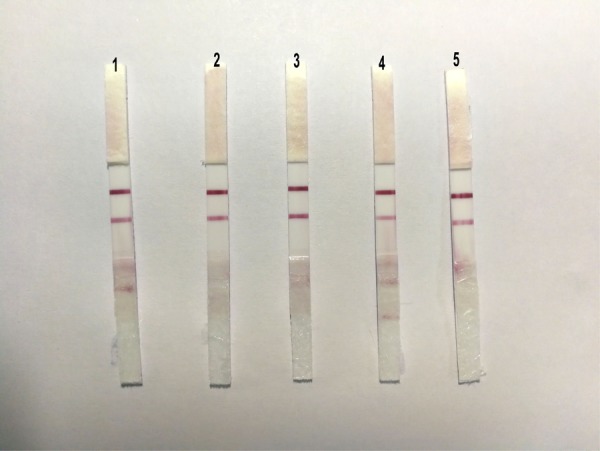
The sensibility of the ICS. The same strain positive allantoic fluid of DTMUV was detected by five different batches of ICS, and there were no obvious differences among the five batches of ICS.

To evaluate the repeatability of ICS, we detected the same strain positive allantoic fluid of DTMUV with different batches of ICS, and the results showed that there were no obvious differences among the five batches of ICS (**Figure 6**).

### Stability

The persistent stability of the ICS is an important consideration for field application. The stability of the antibodies (i.e., A12D3 and C12D1) immobilized on the ICS was tested by storing the assay at 4 and 25°C for 1–10 months. From **Table 2**, the ICS could be stored for 4 months at 25°C and for 6 months at 4°C.

**Table 2:** Stability experiment results from ICS.

Storage temperature	Storage time (month)									
	1	2	3	4	5	6	7	8	9	10
Room temperature (25°C)	+++	+++	+++	+++	++	+	+	–	–	–
Refrigerant temperature (4°C)	+++	+++	+++	+++	+++	++	++	+	–	–

*+++, the valid rate was 100%; ++, the valid rate was 66%; +, the valid rate was 33%; -, the valid rate was 0%, *n* = 5.*

### Practicability

Fifty clinical samples (i.e., 35 clinical samples and 15 positive allantoic fluid samples) were tested in parallel using the ICS and RT-PCR. The results showed that the positive detection rate of both detection methods was 92 [(32+14)/50] and 98% [(34+15)/50], respectively. The coincidence rate of these two methods was 93.9% [(32+14)/(34+15)] (**Table 3**).

**Table 3:** Comparative experimental results from ICS and RT-PCR.

Samples source	Samples	ICS	RT-PCR
Clinical sample	35	32	34
Positive allantoic fluid	15	14	15
Positive detection rate (%)		92	98
Coincidence rate (%)		93.9	

## Discussion

Duck Tembusu virus is a contagious pathogen from fowl that includes ducks and geese with symptoms of high fever, loss of appetite, retarded growth, neurological signs, and severe duck-drop syndrome (Cao et al., 2011). To date, DTMUV has caused an enormous loss to many egg-laying and breeder duck farms in China (Yan et al., 2011). Thus, an efficient method to detect DTMUV is one of the important measures to monitor the spread of this viral contagious disease.

Current approaches for clinical diagnosis of DTMUV infection are mainly classified into three categories: virus isolation, a PCR-based assay, and an immunological method. Examples include virus isolation (Yan et al., 2011), RT-PCR (Cao et al., 2011), real-time PCR (Yun et al., 2012), semi-nested PCR (Tang et al., 2015), loop-mediated isothermal amplification (Tang et al., 2012), blocked ELISA (Li et al., 2012), competition ELISA (Fu et al., 2015), and indirect immunofluorescent assay (IFA) (Wu et al., 2016). The above diagnostic methods have their advantages and disadvantages, regarding sensitivity, accuracy, operability, and cost, and it takes to do them. For example, ELISAs are commonly used for filed detection due to its high sensitivity and specificity (Chen et al., 2014; Wang et al., 2014; Lin, 2015), but it usually requires expensive reagents, special equipment, well-trained staff, several hours, and only be carried out in laboratories. All of these detection methods are inconvenient and not easy enough for field diagnoses or small hospitals. Therefore, to solve these issues, we prepared a rapid and simple diagnostic technique for DTMUV the Colloid Gold Immunochromatographic Assay, which is cheap, easy to prepare, and does not skilled staff or specialized equipment.

In this study, the developed ICS was based on antibodies of E protein of this virus. E protein, as a major envelop protein of flavivirus, has multi-antigenic determinants and plays an important role in mediating the host-related immune response and producing neutralizing antibodies (Allison et al., 2001; Chen et al., 2005, 2014; Mukhopadhyay et al., 2005; Pierson et al., 2008; Chavez et al., 2010; Han et al., 2016). Therefore, we utilized the purified monoclonal antibody A12D3 against the envelop E protein as a gold conjugate antibody, while the preparation of purified polyclonal antibody C12D1 from BALB/c mice against the envelope E protein served as the capture antibody. The specificity and sensibility results showed that the ICS only reacted to DTMUV within 10 min and the diluted test samples were higher as well. Thus, it can be seen that the ICS showed high specificity and sensibility.

The low number of test steps in assay minimizes the risk of contamination, thereby allowing better understanding of outbreaks of the infectious disease (Kim et al., 2007; Nakayama et al., 2014). The rapid and accurate detection is crucial to help determine whether to initiate treatment, and it will also reduce economic losses in the animal industry (Kim et al., 2015). In our study, in order to evaluate how practical for detecting DTMUV, we detected 50 samples with ICS and RT-PCR (Zhang et al., 2015b; Siu et al., 2016; Zeng et al., 2016; Zhao et al., 2016). As expected, the results detected by ICS were generated within 10 min, which was significantly shorter than the time taken to do RT-PCR (about 5 h). Importantly, the results generated by ICS can be read directly with the naked eye. The shorter detection time is essential for monitoring the spread of DTMUV. Moreover, the coincidence rate of these two detection methods reached 93.9%, which implies ICS has better accuracy and reliability for the rapid detection of DTMUV. The long-term stability of ICS is also a critical factor for clinical use. As expected, it could be stored for up to 6 months at 4°C. Hence, ICS has an optimal clinical practicability for its convenience and reliability.

## Conclusion

The present study provided an ICS for the rapid detection of DTMUV. The ICS demonstrated the advantages of high specificity and sensibility, good repeatability, convenience, and clinical application. Above all, it can be easily performed by unskilled staff, and the result is visualized with the naked eye within 10 min. In the future development of viral detection by ICS, quantitative, or semi-quantitative assay, and a multifunctional strip to detect two or more agents will be the next step.

## Ethics Statement

In this study, all experiments were performed according to the guidelines of the Committee on the Ethics of Animals of Shandong and the appropriate biosecurity guidelines. The protocol was approved by Shandong Agricultural University Animal Care and Use Committee (No. SDAUA-2016-099).

## Author Contributions

GYu and GYa completed most of the works. XY designed the tables and figures. YT and YD gave experimental instruction. Thank all the authors’ contribution to the study.

## Conflict of Interest Statement

The authors declare that the research was conducted in the absence of any commercial or financial relationships that could be construed as a potential conflict of interest.
