# Supplementary material for: A Novel Diagnostic Method to Detect Duck Tembusu Virus: A Colloidal Gold-Based Immunochromatographic Assay
Source: Front Microbiol. 2018 May 15;9:1001. doi: 10.3389/fmicb.2018.01001 (PMC5963251; doi:10.3389/fmicb.2018.01001)
Supplement: Supplementary file 1 [file Presentation_1.pdf]

Supplemental Information

In this study, a novel diagnostic ICS assay was developed to detect DTMUV. The assay was carried out using colloidal gold coated with purified mAb-A12D3 against envelope E protein. Purified PaAb-C12D1 from BALB/c mice against the envelope E protein were used as the capture antibody. The specificity and sensitivity of above two antibodies were detected using an indirect ELISA (iELISA)(Bai et al., 2015; Lin, 2015).

From the Figure 1, the mAb-A12D3 and the PcAb-C12D1 were only reacted with the positive serum samples of DTMUV(+), and had no cross reaction with others viruses that can infect ducks (i.e., H9N2 AIV, DPV, NDV, ARV, DHAV-1, EDSV, and MPV).

From the Figure 2, we can see that the sensitivities of mAb-A12D3 and PaAb-C12D1 for DTMUV positive serum dilutions were 1:800 and 1:1600.

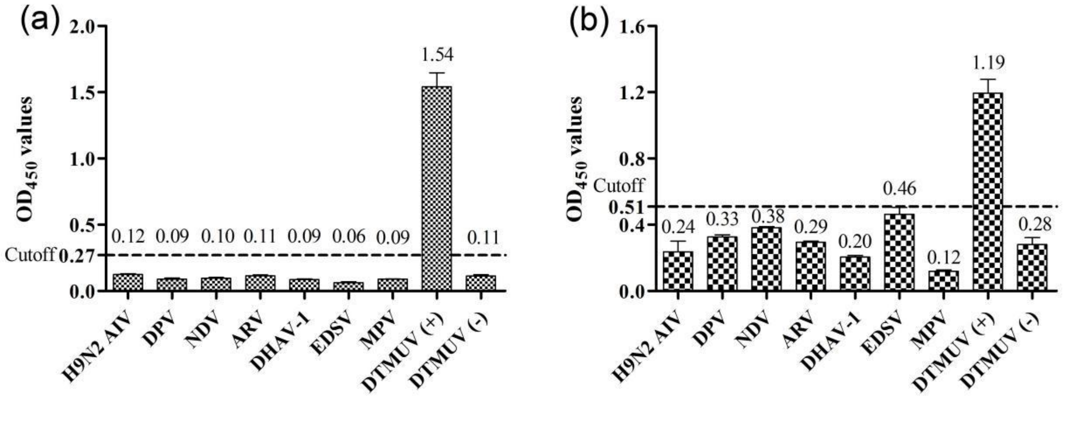

Figure 1. The specificity of mAb-A12D3 (a) and PcAb-C12D1 (b) (*n* = 3). The optical density at 450 nm (OD450) was measured by microplate reader. The values were expressed as Mean±standard deviation (SD). The same as below Figure.

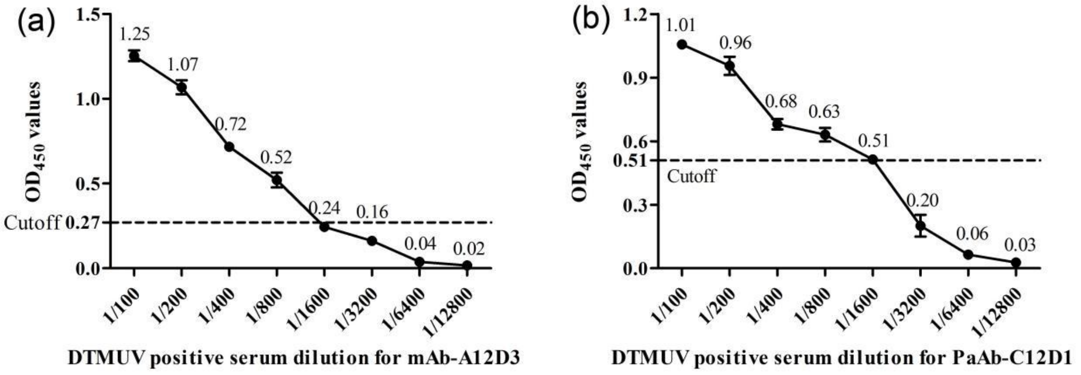

Figure 2. The sensitivity of mAb-A12D3 (a) and PcAb-C12D1 (b) (*n* = 3).
